# Shared and Unique Risk Factors Underlying Mathematical Disability and Reading and Spelling Disability

**DOI:** 10.3389/fpsyg.2016.00803

**Published:** 2016-06-10

**Authors:** Esther M. Slot, Sietske van Viersen, Elise H. de Bree, Evelyn H. Kroesbergen

**Affiliations:** ^1^Department of Education, Faculty of Social and Behavioral Sciences, Utrecht UniversityUtrecht, Netherlands; ^2^Research Institute of Child Development and Education, University of AmsterdamAmsterdam, Netherlands; ^3^Department of Special Education, Utrecht UniversityUtrecht, Netherlands

**Keywords:** reading and spelling disability, mathematical learning disability, comorbidity, multiple deficit model, phonological processing

## Abstract

High comorbidity rates have been reported between mathematical learning disabilities (MD) and reading and spelling disabilities (RSD). Research has identified skills related to math, such as number sense (NS) and visuospatial working memory (visuospatial WM), as well as to literacy, such as phonological awareness (PA), rapid automatized naming (RAN) and verbal short-term memory (Verbal STM). In order to explain the high comorbidity rates between MD and RSD, 7–11-year-old children were assessed on a range of cognitive abilities related to literacy (PA, RAN, Verbal STM) and mathematical ability (visuospatial WM, NS). The group of children consisted of typically developing (TD) children (*n* = 32), children with MD (*n* = 26), children with RSD (*n* = 29), and combined MD and RSD (*n* = 43). It was hypothesized that, in line with the multiple deficit view on learning disorders, at least one unique predictor for both MD and RSD and a possible shared cognitive risk factor would be found to account for the comorbidity between the symptom dimensions literacy and math. Secondly, our hypotheses were that (a) a probabilistic multi-factorial risk factor model would provide a better fit to the data than a deterministic single risk factor model and (b) that a shared risk factor model would provide a better fit than the specific multi-factorial model. All our hypotheses were confirmed. NS and visuospatial WM were identified as unique cognitive predictors for MD, whereas PA and RAN were both associated with RSD. Also, a shared risk factor model with PA as a cognitive predictor for both RSD and MD fitted the data best, indicating that MD and RSD might co-occur due to a shared underlying deficit in phonological processing. Possible explanations are discussed in the context of sample selection and composition. This study shows that different cognitive factors play a role in mathematics and literacy, and that a phonological processing deficit might play a role in the occurrence of MD and RSD.

## Introduction

During these last years, there has been a shift from interpreting developmental learning disabilities as being caused by one single underlying deficit to being the result of multiple (interacting) etiological influences (e.g., Pennington, 2006; McGrath et al., 2011; Van Bergen et al., 2014). The single-deficit model, which assumes that learning disabilities arise from one core underlying deficit, is considered to be too deterministic (Pennington, 2006). In contrast, multiple-deficit models assume that several cognitive weaknesses contribute to the development of a specific learning disability, where some cognitive deficits are seen as unique cognitive risk factors and others are shared between disabilities. These shared risk factors may account for a greater than expected co-occurrence between disabilities, i.e., comorbidity. Multiple-deficit models can therefore be a powerful method to study comorbidity between neurodevelopmental disabilities (see e.g., McGrath et al., 2011; Willcutt et al., 2013).

Despite the increasing attention on multiple-deficit models, relatively few studies have examined possible shared cognitive risk factors between mathematical disability (MD) and reading and spelling disability (RSD). Children with MD experience persistent difficulties with numerosity, especially understanding conceptual properties of numbers and acquiring number fact knowledge (Cirino et al., 2007; Geary, 2013). RSD is defined as a persistent difficulty in acquiring basic reading and/or spelling subskills such as word identification and phonological decoding (Vellutino et al., 2004; Rose, 2009). Comorbidity prevalence rates between MD and RSD are substantial, ranging from 11 to 70% (Lewis et al., 1994; Gross-Tsur et al., 1996; Von Aster et al., 2007; Landerl and Moll, 2010; Moll et al., 2014a) rendering the question of whether there are shared risk factors between the two. The present study includes specific as well as shared cognitive predictors for MD and RSD into a multi-factorial risk model in order to test the extent to which we can account for comorbidity between the two symptom dimensions of math and literacy.

For MD, research has indicated that impairments might exist in WM, leading to difficulty with executing calculation procedures and learning arithmetic facts (e.g., Schuchardt et al., 2008; Geary et al., 2009; Raghubar et al., 2010). In addition, a central deficit in the processing of number magnitude information might be related to MD (i.e., number sense, NS; Wilson and Dehaene, 2007; Landerl et al., 2009; Moeller et al., 2012; Kroesbergen and Van Dijk, 2015). However, whether these number processing deficiencies are specific to symbolic magnitudes (i.e., numbers; Rousselle and Noël, 2007) or also involve non-symbolic magnitudes (e.g., dots; Landerl et al., 2009; Moll et al., 2015) is still debated. Furthermore, some studies have found rapid automatized naming (RAN) to be impaired in children with MD (De Weerdt et al., 2013; Willcutt et al., 2013; Donker et al., 2016), but others have not (e.g., Landerl et al., 2009). RAN is considered to be the ability to access and retrieve phonological representations rapidly from long-term memory (Willburger et al., 2008). Recently, Donker et al. (2016) reported that only non-alphanumeric RAN (i.e., RAN colors and pictures) was impaired in children with MD, but not alphanumeric RAN (i.e., RAN of letters and digits). They hypothesize that children with MD might be impaired in a process called *conceptual* processing (i.e., recalling semantic information from memory), required for non-alphanumeric RAN, but less for alphanumeric RAN, which mainly taps print-to-sound translation processes (access-deficit).

A large body of evidence has indicated specific risk factors related to RSD. Phonological awareness (PA), the ability to recognize and manipulate individual speech sounds (phonemes) and combinations of speech sounds, has been found to be significantly related to the development of RSD (Vellutino et al., 2004). In addition, poorer RAN (Willburger et al., 2008) and reduced verbal short term memory (Verbal STM) capacity (Swanson et al., 2009) have been identified as possible risk factors associated with RSD. Note, however, that the contributions of PA, RAN and Verbal STM can differ between orthographies and ages (e.g., De Jong and Van der Leij, 1999, 2003; Georgiou et al., 2008; Smythe et al., 2008; Landerl et al., 2013; Moll et al., 2014b). Furthermore, the risk factors can contribute differently to reading and spelling (e.g., Moll and Landerl, 2009; Georgiou et al., 2012; Moll et al., 2014c). These findings do not always fully endorse the (universal) presence of these risk factors to the same extent (e.g., Pennington et al., 2012).

Despite the fact that MD and RSD co-occur at a greater-than-chance level, a limited number of studies have systematically examined the overlap between RSD and MD (e.g., Landerl et al., 2004, 2009; Willcutt et al., 2013; Moll et al., 2014c; Cirino et al., 2015; Donker et al., 2016; Peterson et al., 2016). These studies identified risk factors specific to MD (i.e., visuospatial WM, NS) and RSD (i.e., PA), as well as potentially shared risk factors (i.e., WM, processing speed, verbal comprehension, phonological processing; Geary et al., 2000; Willburger et al., 2008; Landerl et al., 2009; Willcutt et al., 2013; Donker et al., 2016). However, many of these studies were focused on a specific sample of children (e.g., twins), or a small set of risk factors (e.g., WM). Here, we contribute to this matter by including multiple specific risk factors for both MD (visuospatial WM, NS) and RSD (alphanumeric and non-alphanumeric RAN, PA, Verbal STM) and by further developing the line of inquiry initiated by Geary (1993), Landerl et al. (2009), and Wilson et al. (2015) on the potential role of phonological processing as a shared risk factor for MD and RSD. In order to maximize variation in the symptom dimensions (math, i.e., fact retrieval and complex math skills, and literacy, i.e., spelling and reading) we tested our multi-factorial (comorbidity) model in a broad sample, including typically developing (TD) children as well as children with MD and/or RSD.

The goal of this study was to assess whether the multiple risk model can account for the comorbidity between MD and RSD by studying the contribution of different cognitive skills to math and literacy outcomes. It was hypothesized that in line with the multiple-deficit view we would find at least one unique predictor for both MD and RSD and a possible phonological processing measure that can partly account for the comorbidity between the two symptom dimensions (i.e., RAN or PA). In relation to model testing, we hypothesized that (a) a multi-factorial risk factor model would provide a better fit to the data than a single risk factor model and (b) a shared risk factor model would provide a better fit than a multi-factorial risk factor model. On the basis of findings that there might be differences between alphanumeric and non-alphanumeric RAN in terms of the strength of associations with literacy (van den Bos et al., 2002) and differences in breadth of the RAN-deficit (Donker et al., 2016), RAN was divided into an alphanumeric and non-alphanumeric component, which were added to the model as two distinct predictors. Structural equation modeling (SEM) was applied, as this has been proposed to be an appropriate method for testing multiple-deficit models (e.g., Pennington et al., 2012; Peterson et al., 2016).

## Materials and methods

### Participants

Participants included 130 7-to-10-year-old Dutch primary school children (37.2% boys), with a mean age of 8;10 years (*SD* = 12 months). All children attended primary schools in the Netherlands (Grade 1 through 5), with the majority (95.5%) in Grades 2, 3, and 4. Recruitment took place through advertisements on special educational needs websites, or contacts with specialized clinical centers and schools. Informed consent was obtained from all participants and their parents before testing. The mean IQ score for the total sample was 102.00 (*SD* = 10.44). Children were included in the sample based on a screening by a clinical expert, following criteria in line with current diagnostic criteria in the Netherlands for MD and RSD. Based on their test scores, dossier information about diagnoses, and received help, children were divided into four groups: a typically developing (TD), reading and/or spelling difficulty (RSD), mathematical difficulty (MD), and a comorbid (RSD+MD) group. Children were considered to have MD if they obtained basic arithmetic scores of 1SD below the mean of the TD children group as well as scored at or below the 25th percentile on a math problem solving test (D/E scores; cf. Janssen et al., 2010). Moreover, MD children should show average scores (standard score ≥ 8 or percentile ≥ 25) on reading and spelling measures. Children were classified as having RSD if they scored 1SD below the population mean on word or pseudoword reading and/or achieved a score at or below the 10th percentile on a spelling test administered at school (E score) (cf. Kuijpers et al., 2003; Kleijnen et al., 2008), but showed average arithmetic performance (standard score ≥ 8 or percentile ≥ 25). Children with comorbid difficulties had to meet both the MD and RSD requirements. TD children had to show average reading, spelling, arithmetic, and mathematics performance (standard score ≥ 8 or percentile ≥ 25). All children had to have an IQ between 80 and 125, and no reported history of sensory impairment, serious emotional or behavioral problems, or developmental disabilities (e.g., ADHD, autism spectrum disorder).

Descriptive statistics for all behavioral and cognitive measures in every group are displayed in Table 1. In total, 26 children were included in the MD group, 29 children in the RSD group, 43 children met criteria for both RSD and MD and 32 children were included in the TD control group.

**Table 1:** **Descriptive measures for the total sample (*N* = 130) and TD, RSD, MD, and RSD+MD groups**.

	**TD (*n* = 32)**		**RSD (*n* = 29)**		**MD (*n* = 26)**		**RSD+MD (*n* = 43)**	
**Measures**	***M***	***SD***	***M***	***SD***	***M***	***SD***	***M***	***SD***
Age	103.34^a^	8.79	104.31^a^	12.45	106.69^a^	11.89	113.84^b^	10.42
Full scale IQ	108.62^a^	9.53	105.15^ab^	10.02	99.79^b^	9.34	96.50^c^	8.71
Timed reading test	55.16^a^	10.57	32.58^b^	13.00	57.17^a^	14.04	37.79^b^	12.84
Timed non-word reading	47.13^a^	13.73	21.42^b^	10.67	46.45^a^	14.99	27.72^b^	11.95
Spelling to dictation	74.44^a^	21.68	55.88^b^	22.86	75.82^a^	20.90	56.16^b^	18.51
**MATH ABILITY**								
Addition	18.72^a^	4.39	16.92^ab^	5.94	14.45^b^	4.40	14.79^ab^	4.26
Subtraction	16.25^a^	3.91	13.92^ac^	5.48	10.21^b^	4.44	10.88^bc^	4.46
Multiplication	14.76^a^	5.13	13.86^ab^	7.21	9.83^b^	6.07	10.50^ab^	5.24
Division	8.79^a^	4.64	7.95^a^	6.25	4.07^b^	3.39	4.24^b^	3.51
Mathematical problem solving	64.57^a^	19.29	61.73^a^	24.53	45.38^b^	25.49	53.41^ab^	21.87
**RAPID NAMING**								
Colors	48.19^a^	9.31	58.23^bc^	15.01	52.00^ab^	10.88	55.33^ac^	12.78
Pictures	51.69^a^	8.11	61.92^bc^	15.24	52.48^ab^	10.86	56.40^ac^	11.34
Letters	30.00^a^	6.18	37.65^bc^	11.23	30.79^ab^	7.79	35.00^ac^	10.76
Digits	30.09^a^	6.68	33.96^a^	7.65	28.69^a^	5.40	31.70^a^	6.17
**PHONEME AWARENESS**								
Phoneme manipulation	0.08^a^	0.048	0.031^b^	0.034	0.048^ab^	0.043	0.030^b^	0.025
Phoneme deletion	0.32^a^	0.131	0.201^b^	0.153	0.256^b^	0.123	0.190^b^	0.094
**VERBAL SHORT TERM MEMORY**								
Digit Recall	24.61^a^	4.26	23.88^a^	3.49	24.45^a^	2.56	22.97^a^	4.48
Word Recall	24.13^a^	2.74	23.96^a^	3.56	24.41^a^	3.36	23.62^a^	3.03
**VISUAL-SPATIAL WORKING MEMORY AND NUMBER SENSE**								
Dot Matrix	21.45^a^	5.46	21.12^a^	5.69	18.59^a^	3.54	20.95^a^	4.84
Spatial Span	16.45^a^	4.55	14.62^a^	6.49	13.59^a^	4.58	13.51^a^	5.59
Odd One Out	16.94^a^	3.43	15.77^a^	5.58	14.00	4.36	15.13^a^	4.71
Number line estimation (R^2^)	0.95^a^	0.034	0.882^a^	0.178	0.820^b^	0.171	0.878^ab^	0.127

*TD, typically developing; RSD, reading/spelling disabilities; MD, mathematical disabilities; RSD+MD, comorbid group; Group means with the same superscripts do not differ (p < 0.05)*.

### Instruments

#### Reading

Timed (pseudo)word reading measures were used, taking both word reading accuracy and fluency into account. The Eén Minuut Test (EMT; Brus and Voeten, 1999) consists of a columned list of 116 unrelated (existing) words, increasing in length from one to four syllables. Participants were instructed to fastly read aloud as many words as they could, without making errors. The number of words read correctly in 1 min was computed. The Klepel (Van den Bos et al., 1994) consists of 116 pseudowords, which are similar to the structure of Dutch words (as in EMT) and of increasing complexity. Instruction was identical to the EMT, although the time limit was 2 min. Again, the test score was the amount of pseudowords read correctly in 2 min. Reliabilities were 0.91 for the EMT and 0.92 for the Klepel (Evers et al., 2009–2012).

#### Spelling

Spelling was assessed using a shortened version of a spelling to dictation task (PI dictee; Geelhoed and Reitsma, 1999), including 42 words (6 sets of 7 words; P. F. de Jong, personal communication, September 2012). The task included regularly spelled words, words containing spelling rules, and irregular words. The test was stopped after children spelled five or more words incorrectly within one set. The internal consistency of the full version varied between 0.90 and 0.93 (Evers et al., 2009–2012).

#### Math ability

A speeded arithmetic test, Tempo Toets Rekenen (TTR; De Vos, 1992) was used to measure children's timed arithmetic ability. For each subtest, children were instructed to solve as many problems as they could in 1 min. The first subtest required addition, followed by subtraction, multiplication, and division. Every subtest included 40 problems of increasing complexity. Cronbach's alpha was 0.86 for the addition and subtraction scale and 0.83 for multiplication and division scale.

The national norm-referenced CITO mathematics test was used to measure mathematical problem solving (Janssen et al., 2010). The test has different items for different age groups. Test scores are converted into normed “ability scores,” provided by the publisher, that typically increase throughout primary school, allowing a comparison of results throughout the academic career (Janssen et al., 2005). The CITO mathematics test has been shown to be highly reliable; coefficients of different versions range between 0.91 and 0.97 (Janssen et al., 2010).

#### Intelligence

To assess children's cognitive ability, a short form of the Dutch version of the Wechsler Intelligence Scale for Children NL (WISC-III-NL; Kort et al., 2005) was used, consisting of the verbal subtests Similarities and Vocabulary and the performance subtests Picture Completion and Block Design. The reliability and validity quotients of this short form are all reported to be above 0.83 (Kaufman et al., 1996).

#### Phonological awareness

The Dutch Fonemische Analyse Test (FAT; Van den Bos et al., 2009) is a timed computerized test consisting of two subtests: Phoneme Deletion (PD) and Phoneme Manipulation (PM). PD demanded children to repeat a word and delete the initial, middle or last sound (e.g., boek “book” without /b/ is oek). PM required children to switch the first sounds of two given words (e.g., Moeder Gans “Mother Goose” becomes Goeder Mans). Raw accuracy score and online computed reaction times were transformed into the number of correct responses per second. Internal consistency of the total test is reported to be 0.93 (Evers et al., 2009–2012).

#### Rapid automatized naming

The Continu Benoemen and Woorden Lezen test (CB and WL; Van den Bos and Lutje Spelberg, 2007) includes rapid naming of letters (s, p, a, d, o), digits (2, 4, 8, 5, 9), pictures (bicycle, tree, chair, duck, scissors) and colors (black, green, yellow, red, green). Children were instructed to name the visually presented information as quickly as possible without making mistakes. Raw scores (time in seconds) were used. Split-half reliability varied between subtests from 0.82 to 0.90 (Evers et al., 2009–2012).

#### Memory

Subtests of the Automated Working Memory Assessment (AWMA; Alloway, 2007) were used to assess the different memory components. Verbal STM was measured using the digit recall and word recall subtests. For visuospatial WM, dot matrix, spatial span, and odd one out subtests were used. All tasks correspond to the Baddeley WM model (1986). Per subtest, testing was terminated after three incorrect responses. Raw scores (i.e., number of correct items) were used in the analyses. A description of the tasks as well as subtest reliabilities can be found in Alloway et al. (2009).

#### Number sense

NS was assessed with the number line estimation task reported in Kolkman et al. (2013). This task demanded children to indicate where the researcher should place a lever on a number line from 0 to 100 to position a presented digit. The proportion of explained variance (R^2^) was computed by fitting the answers of each child on a linear curve (see also Kolkman et al., 2013). The task was administered on a laptop computer using E-prime 1.2 software (Psychological Software Tools, http://pstnet.com). Internal consistency of the test was 0.79 (Kolkman et al., 2013).

### Procedure

All children were tested individually by a trained and supervised graduate student in a quiet room at school or at home. The neuropsychological and behavioral test battery comprised 2.5 to 3 h, depending on whether intelligence measures were available, with ample breaks between tasks. Parents and schools could indicate whether they wanted a test report children received a reward (i.e., a sticker) after every test they completed. For this study, data from largely the same set of participants was used as is in Donker et al. (2016). The IQ range was limited to 80–125, excluding three participants with an IQ > 125. Hence, whereas the total sample of Donker et al.'s study included 133 students, our study included 130 participants. This resulted in slightly weaker correlations between the math and literacy outcome variables and the RAN measures, although the *p*-values remained similar.

### Data analysis

Correlational analyses revealed that for some of the variables performance increased as a linear function of age. These variables (EMT, PI-dictation, TTR, CITO math, NS, RAN letters and RAN numbers) were transformed into age-residualized scores by regressing the variable on age and age squared and saving the unstandardized residuals (see also McGrath et al., 2011). The PM task results were log-transformed in order to approximate a normal distribution. Outliers (*z*-scores > 3.29 or < −3.29) were removed from the data.

Confirmatory Factor Analyses (CFAs) and SEM were performed in Mplus version 6.12 (Muthén and Muthén, 2007). Maximum likelihood estimation with robust standard errors (i.e., MLR) was used to deal with non-normality in some of the variables and avoid listwise deletion. Missing data was minimal for both the behavioral and the cognitive measures (0–10%) and handled using full information maximum likelihood (FIML) estimation.

In order to test our hypotheses, a four-step approach was taken to build toward a comorbidity model. First, CFAs were run on the continuously distributed symptom (i.e., math and literacy) and cognitive dimensions (NS, visuospatial WM, PA, RAN, Verbal STM) separately. In these measurement models, the latent factors represented the continuously distributed symptoms of MD and RSD. Second, a single risk factor model was tested, in which one deterministic risk factor for both disabilities was regressed on literacy and math. Based on evidence from previous empirical studies on the etiology of MD and RSD and correlational analyses (Table 2), NS and PA were selected as the specific cognitive risk factors for these analyses. Third, a multi-factorial specific risk factor model was tested in which NS, visuospatial WM, PA, RAN, and Verbal STM were all included as specific risk factors for the individual difficulties. Fourth, a comorbidity model was tested in which a shared risk factor was added to the multi-factorial model. Satorra-Bentler chi-square difference tests were used to compare model fit of all three SEM models. The following criteria for model evaluation were used: chi-square value (χ^2^) with associated *p*-value, RMSEA including *p*close, CFI, and SRMR (Kline, 2011; Little, 2013). For good model fit, chi-square should have a non-significant *p*-value (i.e., >0.05), RMSEA should be < 0.05 (< 0.08 is acceptable), with *p*close >0.05, CFI being >0.95 (>0.90 is acceptable), and SRMR being < 0.05 (< 0.08 is acceptable; Kline, 2011; Little, 2013).

**Table 2:** **Correlations between symptom and cognitive dimensions corrected for age**.

	**FAT**		**Number sense**	**Verbal STM**		**Visuospatial WM**			**RAN**			
**Variable**	**PD**	**PM**	***r*^2^**	**DR**	**WR**	**SS**	**DM**	**OOO**	**Colors**	**Digits**	**Pictures**	**Letters**
EMT	0.49^**^	0.44^**^	0.14	0.09	0.06	0.18	0.01	0.22^*^	−0.28^**^	−0.50^**^	−0.44^**^	−0.65^**^
Klepel	0.50^**^	0.47^**^	0.05	0.11	0.05	0.14	0.01	0.19	−0.34^*^	0.50^**^	−0.42^**^	−0.58^**^
PI-dictee	0.59^**^	0.62^**^	0.15	0.14	0.11	0.26^*^	0.16	0.26^*^	−0.27^**^	−0.30^**^	−0.30^**^	−0.46^**^
TTR +	0.25^*^	0.36^**^	0.36^**^	0.03	0.11	0.30^**^	0.38^**^	0.27^**^	−0.13	−0.28^**^	−0.26^**^	−0.25^*^
TTR -	0.31^**^	0.37^**^	0.41^**^	−0.02	0.10	0.38^**^	0.37^**^	0.28^**^	−0.22^*^	−0.17	−0.16	−0.10
TTR x	0.18	0.31^**^	0.32^**^	−0.14	−0.08	0.22^*^	0.23^*^	0.15	−0.23^*^	−0.19	−0.25^*^	−0.22^*^
TTR:	0.28^**^	0.41^**^	0.27^**^	−0.01	0.15^**^	0.34^**^	0.34^**^	0.34^**^	−0.31^**^	−0.16	−0.20^*^	−0.12
Cito	0.28^**^	0.40^**^	0.30^**^	0.17	0.19	0.38^**^	0.31^**^	0.36^**^	−0.28^**^	−0.00	−0.12	−0.21^*^

*EMT, word reading; Klepel, nonword reading; PI dictee, spelling task; TTR, speeded arithmetic task; Cito, mathematics task; FAT, phonological awareness task; STM, short-term memory; WM, working memory; RAN, rapid automatized naming; PD, phoneme deletion; PM, phoneme manipulation; DR, digit recall; WR, word recall; SS, spatial span; DM, dot matrix; OOO, odd one out*.

* *p < 0.05*,

** p < 0.01;

*N = 94*.

## Results

Preliminary correlational analyses were conducted on the raw scores, while correcting for age in months, in order to assess whether the cognitive variables were associated with literacy and math outcomes (Table 2). The math ability tasks correlated significantly with the NS, visual-spatial WM, and PA measures, and to a lesser extent with the RAN measures. Correlations between literacy and the cognitive measures for PA and RAN were significant (see Table 2 for detailed information).

### Measurement models

#### Symptom dimensions

**Figure A1:**
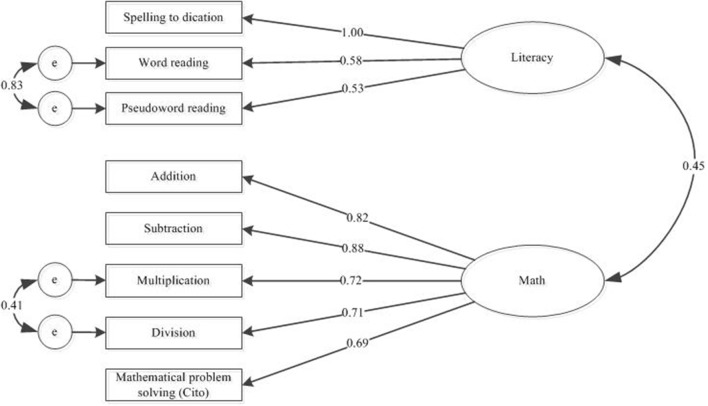
**Measurement model for the symptom dimensions literacy and math**. Standardized path coefficients are depicted.

The measurement model for the symptom dimensions of MD and RSD (with literacy and math ability as continuously distributed outcomes) was first fitted to the data. An error correlation between word reading and pseudoword reading as well as between the multiplication and division scores were allowed after consulting the Modification Indices. The residual variance of the spelling measure was set to zero since it was not significant. After these adjustments, the proposed model showed a good fit, χ^2^ (18, *n* = 130) = 18.85, *p* = 0.40, RMSEA = 0.02, 90% Confidence Interval (CI) = [0.00 – 0.08], *p*close >0.05, CFI = 1.00, SRMR = 0.03. A depiction of the measurement model for the MD and RSD symptoms is included in the Appendix, Figure A1.

#### Cognitive dimensions

**Figure A2:**
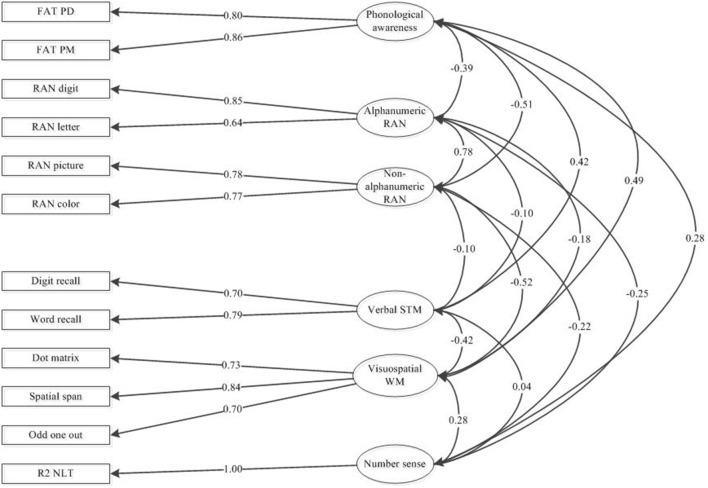
**Measurement model for the cognitive dimensions NS, visuospatial WM, PA, RAN, and verbal STM**. Standardized path coefficients are depicted.

The measurement model for the continually distributed cognitive dimensions (NS, visuospatial WM, PA, alphanumeric and non-alphanumeric RAN, and Verbal STM) fitted the data well, χ^2^ (40, *n* = 130) = 49.87, *p* = 0.14, RMSEA = 0.04, 90% CI = [0.00 − 0.08], *p*close > 0.05, CFI = 0.98, SRMR = 0.05. The residual variance of the single indicator for the NS latent variable was fixed to zero. A figure depicting the measurement model for the cognitive dimensions is included in the Appendix, Figure A2.

### Structural equation models

**Figure A3:**
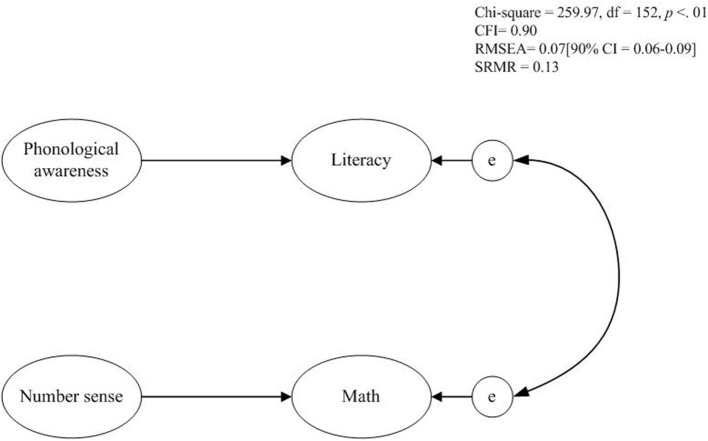
**Single risk factor model**. Standardized path coefficients are depicted.

**Figure A4:**
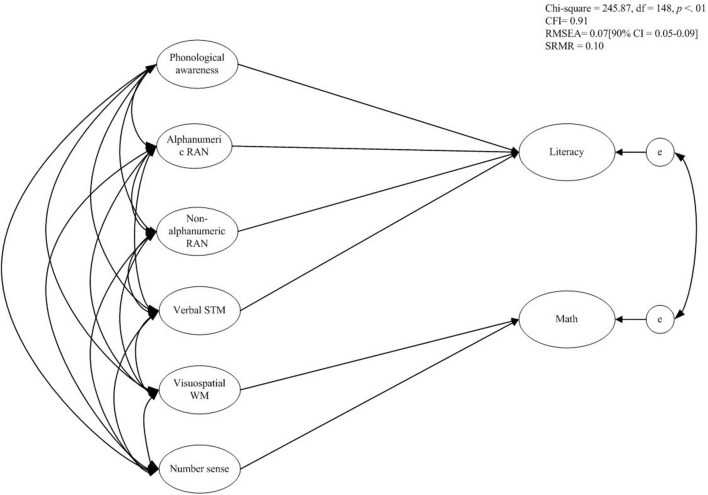
**Multi-factorial risk factor model**. Standardized path coefficients are depicted.

The measurement models for the cognitive and symptom dimensions were combined and structural relations were included in the model equations in order to create a SEM. Three (nested) models were fitted to the data: a single risk factor model, a multi-factorial risk factor model and a comorbidity (shared risk factor) model, in order to test the hypothesis that the latter model most adequately explains the MD/RSD symptoms in this sample. Depictions of the first two models are included in the Appendix: Figures A3, A4.

#### Single risk factor model

A deterministic, single risk factor model was fitted to the data with NS as a risk factor for MD and PA as a risk factor for RSD. This model indicated a just sufficient fit to the data, with χ^2^ (152, *n* = 130) = 259.97, *p* < 0.01, RMSEA = 0.07, 90% CI = [0.06 − 0.09], *p*close < 0.05, CFI = 0.90, SRMR = 0.13. NS was a significant predictor for MD (β = 0.45) and PA for RSD (β = 0.61). In total, NS explained 21% of the variance in the children's math ability and PA explained 38% of the variance in children's literacy (reading and spelling) ability.

#### Multi-factorial risk model

A probabilistic, multi-factorial risk factor model was fitted in order to compare it to the single risk factor model. This model included the following specific risk factors: for math ability, we included NS and visuospatial WM as risk factors for MD. For literacy, we included PA, alphanumeric and non-alphanumeric RAN, and Verbal STM as potential risk factors for RSD. The model fit was considered sufficient, χ^2^ (148, *n* = 130) = 245.87, *p* < 0.01, RMSEA = 0.07, 90% CI = [0.05 – 0.09], *p*close < 0.05, CFI = 0.91, SRMR = 0.10. Of the proposed risk factors for math ability, both NS (β = 0.35) and visuospatial WM (β = 0.40) were significant predictors. For literacy, PA predicted the reading and spelling outcomes significantly (β = 0.70), as well as alphanumeric and non-alphanumeric RAN (β = −0.29 and β = 0.30). Verbal STM was not significantly related to literacy (β = −0.03). A Satorra-Bentler scaled Chi-square difference test indicated that the probabilistic, multi-factorial risk model fitted the data better than the deterministic, single risk factor model, $\chi_{\left(4\right)}^{2}=14.101$, *p* < 0.01. The specific risk factors together explained 36% of the variance in children's math ability and 44% in their literacy scores.

#### Comorbidity model

**Figure 1:**
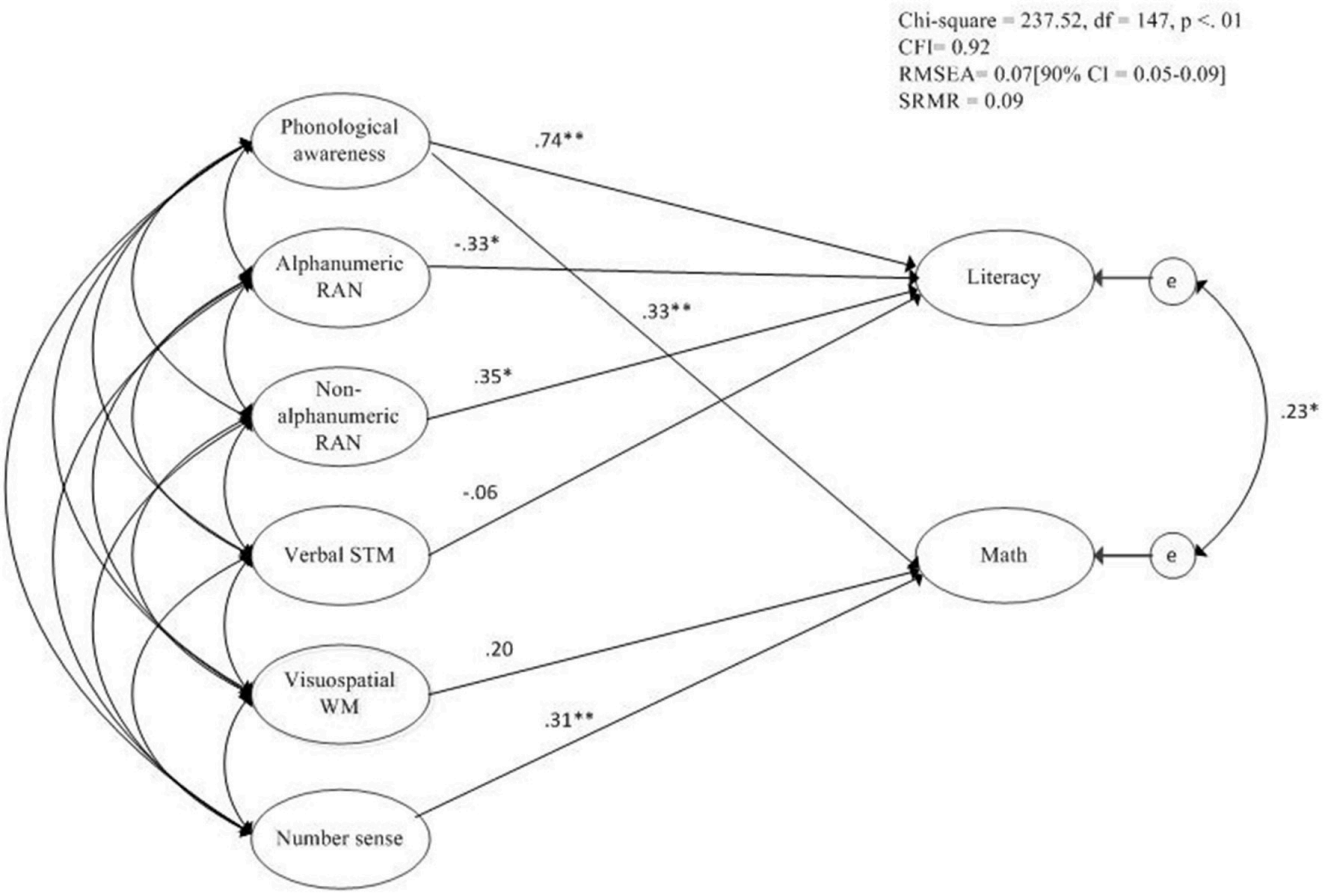
**Comorbidity model including unique and shared risk factors for both MD and RSD**. ^*^p < 0.05; ^**^p < 0.01.

In order to test the proposed shared etiology between MD and RSD, we included PA, alphanumeric RAN, and non-alphanumeric RAN successively as potential shared risk factors in the multi-factorial risk factor model. The model with PA as a shared risk factor fitted the data best, χ^2^ (147, *n* = 130) = 237.52, *p* < 0.01, RMSEA = 0.07, 90% CI = [0.05 – 0.09], *p*close < 0.05, CFI = 0.92, SRMR = 0.09. The comorbidity model is depicted in Figure 1. A Satorra-Bentler Chi-square difference test indicated that the less restricted model (shared risk factor model) provided a better fit to the data than the multi-factorial risk factor model, $\chi_{\left(1\right)}^{2}=8.06$, *p* < 0.01. PA was identified as a shared risk factor (β = 0.34 for math and β = 0.74 for literacy). In total, the cognitive predictors explained 41% of the variance in the children's math ability and 48% of the variance in children's literacy (reading and spelling) ability. The symptom dimensions of MD and RSD were still significantly related, but the relation weakened after adding PA to the model (from β = 0.30 to β = 0.23).

## Discussion

In order to explain the high comorbidity rates between mathematical learning disability (MD) and reading and spelling difficulties (RSD), 7-to-10-year-old Dutch primary school children were assessed on a wide range of cognitive skills related to math and literacy. Following the line of research using multi-factorial risk models, both specific and shared risk factors for MD and RSD were anticipated. Specifically, we expected (a) to find at least one unique predictor for both MD and RSD separately, and a possible shared phonological processing-related risk factor (partly) accounting for the comorbidity between the two. We also hypothesized that (b) a multi-factorial risk factor model would provide a better fit to the data than a single risk factor model and (c) a shared risk factor model would provide a better fit than a multi-factorial risk factor model. All three hypotheses were confirmed.

The results of our study clearly support the multiple-deficit framework proposed by Pennington (2006) in that MD and RSD can be considered two separate but correlated disabilities (Willcutt et al., 2013). In line with previous research, visual-spatial working memory (visuospatial WM) and number sense (NS) were found to be uniquely associated with math ability, constituting specific risk factors for MD (e.g., Schuchardt et al., 2008; Landerl et al., 2009). Similarly, phonological awareness (PA) was a unique predictor of literacy, constituting a specific risk factor for RSD, as has been shown in the literature (e.g., Vellutino et al., 2004; Hulme and Snowling, 2014). Verbal STM did not predict literacy, which can be aligned with findings that the influence of Verbal STM decreases over time as the influence of PA increases (De Jong and Van der Leij, 1999, 2003). In line with the literature, rapid automatized naming (RAN) was also a significant risk factor related to literacy (Van den Bos et al., 2003; Melby-Lervåg et al., 2012; Norton and Wolf, 2012; Protopapas et al., 2013). An important result of the current study is that we found a significant association between NS and MD. More specifically, we used a numberline estimation task measuring the ability to map numbers to “space” (Kolkman et al., 2014). The task however also may require some other forms of strategy use, e.g., proportion judgment (Slusser et al., 2013). Still, our study confirmed that the ability to place numbers on a line seems an important predictor of MD. Another important finding was that we identified PA as a shared cognitive risk factor for MD and RSD; the comorbidity model better fitted the data and explained more variance in both literacy and math performance than the multi-factorial risk factor model without any shared risk factors. These results suggest that MD and RSD co-occur due to a shared underlying deficit (Willcutt et al., 2013). Previous research has suggested the possibility of a phonological processing deficit as a shared risk factor underlying MD and RSD symptoms, but little evidence has been found thus far (Landerl et al., 2009; Wilson et al., 2015).

That PA was identified as a shared risk factor, indicates that phonological skills not only play a role in reading and spelling, but also in mathematics. This supports the findings by Lopes-Silva et al. (2016) that phoneme awareness relates to both word reading and spelling as well as number reading and writing in typically developing children. It also relates to findings by Simmons and Singleton (2007) that the phonological processing deficits of children with dyslexia impair aspects of mathematics that involve the manipulation of verbal codes (e.g., counting speed, number fact recall) and is consistent with the finding that children with dyslexia and mathematical problems often have slow and inaccurate number fact retrieval (Geary et al., 2000). These difficulties with basic arithmetic skills may impact more advanced mathematics directly and indirectly.

Alternatively, the finding of PA as a shared risk factor could indicate that individuals with comorbid MD and RSD might represent a verbal subtype of MD (Geary, 2004; Moll et al., 2015). Researchers have suggested that MD children with difficulties in arithmetic fact retrieval were found to have weaknesses in symbolic number processing (Wilson and Dehaene, 2007; Geary, 2010). This is taken to reflect an access deficit (Skagerlund et al., 2016), relating to problems with accessing the verbal codes of numerical information, requiring phonological processing (Hecht et al., 2001). This could explain the association between PA and math ability. Vice versa, the PA deficit in RSD children could impair aspects of mathematics that involve the manipulation of verbal codes (e.g., counting speed, number fact recall; Simmons and Singleton, 2007). PA could thus be a factor related to verbal codes and subsequent slow and inaccurate number retrieval. It is deemed important that future research further investigates the association between phonological processing and (comorbid) RSD and MD.

An alternative explanation for our finding is that the PA tasks in our study required executive functioning (EF), particularly the phoneme manipulation task were children have to blend and segment words. This “spoonerism” task according to Landerl and Wimmer (2000) includes not only phonological awareness, but also complex memory and monitoring skills. Hence, EF could play a role in the association between PA and MD/RSD rather than phonological awareness itself. However, it must be stressed that the other PA task (phoneme switching) to a much lesser extent appeals to EF and that the PA tasks used in our study are also applied in clinical practice. Nonetheless, it is a serious limitation of the current study that no measures were included on executive functioning (e.g., attentional control, inhibition). Previous research has suggested associations between attention problems and processing speed and comorbid RSD/MD (Willcutt et al., 2013; Moll et al., 2014c; Peterson et al., 2016). However, results from these studies are not unequivocal: associations between executive functions and MD/RSD symptoms were not robust. Future research might therefore try to adopt the multiple-deficit view to individual cases, in order to gain more insight into the clinical utility of these models for explaining comorbidity between RSD and MD (Pennington et al., 2012).

In general, this study has shown that a multiple-deficit framework is suitable for testing shared etiological influences in neurodevelopmental disabilities, but also illustrated the complexity of including multiple unique and shared risk factors into one multiple risk factor model. Although the present study included a wide range of cognitive risk factors, these factors only accounted for 41% of the variance in the MD symptoms and 48% of the RSD symptoms. For example, domain-general factors such as verbal comprehension and processing speed were previously found to be responsible for overlap between behavioral outcomes of math and literacy (e.g., Willcutt et al., 2013; Peterson et al., 2016). Future research could focus on including more domain-general candidate shared risk factors, such as attentional control (Geary, 2013) and executive functioning (i.e., updating; Van der Ven et al., 2012). Also, more specific risk factors that are supposedly uniquely associated with MD and RSD can be included, such as (non-)symbolic comparison skills for MD (Toll et al., 2015) and visual attention span for RSD (VAS; Valdois et al., 2012; Van den Boer et al., 2015). Theoretically and clinically, it is important to account for both MD and RSD as well as the comorbidity between the two. Our study is a stepping stone for future studies in this field.

## Author contributions

ES is responsible for the analyses and the story line in the introduction. SV and ED contributed equally to the manuscript. EK had a supervising role. ES and SV together gathered the data in 2013, supervised by ED and EK.

### Conflict of interest statement

The authors declare that the research was conducted in the absence of any commercial or financial relationships that could be construed as a potential conflict of interest.
